# Development of a Rapid, Simple Method for Detecting *Naegleria fowleri* Visually in Water Samples by Loop-Mediated Isothermal Amplification (LAMP)

**DOI:** 10.1371/journal.pone.0120997

**Published:** 2015-03-30

**Authors:** Aongart Mahittikorn, Hirotake Mori, Supaluk Popruk, Amonrattana Roobthaisong, Chantira Sutthikornchai, Khuanchai Koompapong, Sukhontha Siri, Yaowalark Sukthana, Duangporn Nacapunchai

**Affiliations:** 1 Department of Protozoology, Faculty of Tropical Medicine, Mahidol University, Bangkok, Thailand; 2 Section of Bacterial Pathogenesis, Graduate School of Medical and Dental Sciences, Tokyo Medical and Dental University, Tokyo, Japan; 3 Department of Microbiology, Graduate School of Medicine, Kyoto University, Kyoto, Japan; 4 Department of Epidemiology, Faculty of Public Health, Mahidol University, Bangkok, Thailand; 5 Department of Parasitology, Faculty of Medical Technology, Mahidol University, Bangkok, Thailand; New York University, UNITED STATES

## Abstract

*Naegleria fowleri* is the causative agent of the fatal disease primary amebic meningoencephalitis. Detection of *N*. *fowleri* using conventional culture and biochemical-based assays is time-consuming and laborious, while molecular techniques, such as PCR, require laboratory skills and expensive equipment. We developed and evaluated a novel loop-mediated isothermal amplification (LAMP) assay targeting the virulence-related gene for *N*. *fowleri*. Time to results is about 90 min and amplification products were easily detected visually using *hydroxy naphthol blue*. The LAMP was highly specific after testing against related microorganisms and able to detect one trophozoite, as determined with spiked water and cerebrospinal fluid samples. The assay was then evaluated with a set of 80 water samples collected during the flooding crisis in Thailand in 2011, and 30 natural water samples from border areas of northern, eastern, western, and southern Thailand. *N*. *fowleri* was detected in 13 and 10 samples using LAMP and PCR, respectively, with a Kappa coefficient of 0.855. To the best of our knowledge, this is the first report of a LAMP assay for *N*. *fowleri*. Due to its simplicity, speed, and high sensitivity, the LAMP method described here might be useful for quickly detecting and diagnosing *N*. *fowleri* in water and clinical samples, particularly in resource-poor settings.

## Introduction

Although many species of *Naegleria* have been described, only *N*. *fowleri* has been associated with human disease [1]. It is a free-living protozoan pathogen widely distributed in nature, which can cause a rapidly fatal disease of the central nervous system called primary amebic meningoencephalitis (PAM). *N*. *fowleri* presents three morphological stages, namely the trophozoite, flagellate, and cyst stages. Under favorable conditions, the vegetative trophozoites multiply and feed in the environment. However, under nutrient-limiting conditions, the trophozoite can transform into a non-feeding flagellated form which swims to the water surface and transforms into the ameboid trophozoite to feed on bacteria [2]. The trophozoites can also transform into a cyst stage to protect themselves from harsh conditions, such as food deprivation and desiccation. Due to the free-living nature of *N*. *fowleri*, PAM is generally acquired while swimming and diving in freshwater lakes or ponds where the parasite is splashed or inhaled into the nasal passage, before migrating via the olfactory nerve into the brain [1]. It has been suggested that the trophozoite, the only infectious form, displays a prominent sucker-like structure that is used to ingest bacteria and yeast in the environment as well as brain tissue during infection [3]. PAM has a short incubation period of normally 2 to 5 days and infection usually results in death within 3–7 days after the onset of symptoms [1]. As a consequence, the majority of patients die before they are able to receive appropriate clinical intervention. Therefore, efficient, rapid and timely diagnosis is crucial in order to start treatment due to the aggressiveness of the amoeba. A major problem of PAM is that symptoms of the disease are similar to and often misdiagnosed as bacterial meningoencephalitis, resulting in incorrect management [4, 5]. Conventional diagnosis usually relies on microscopic examination followed by cultivation and confirmation of *N*. *fowleri* in the cerebrospinal fluid (CSF). Morphological criteria, however, are inadequate for distinguishing *N*. *fowleri* from nonpathogenic *Naegleria* spp. and other free-living amoebae. Immunological tests based on rise in antibody titer are also not helpful as PAM progresses rapidly, meaning this method usually provides only late or post-mortem diagnosis [6].

Water supplies can be potential sources of contamination in public and private swimming pools [7], and we likely encounter *N*. *fowleri* during our normal everyday life involved with water. Therefore, an investigation of the occurrence and distribution of *N*. *fowleri* in local water is important because of its possible public health implications and epidemiological studies, e.g., identifying sources of recent infection or risk assessment [8]. To detect *N*. *fowleri* in environmental samples, water samples must be concentrated and cultured on non-nutrient agar plates. This requires an incubation period of at least 48 h on non-nutrient agar, followed by subcultures and identification of *Naegleria* spp. using mouse pathogenicity tests, immunological or biochemical tests [9]. For these reasons, the current diagnosis of infection and identification of *N*. *fowleri* remains unsatisfactory, because culture and mouse pathogenicity are time-consuming, expensive, laborious, and prone to ethical issues. Fortunately, there has been a considerable effort to develop a more reliable and efficient technique for the rapid diagnosis of PAM. Due to advancements in molecular detection, PCR and real-time PCR have been developed and seem to be the most sensitive methods for the rapid identification of *N*. *fowleri* in environmental and clinical samples [6–11]. Despite the high efficiency of PCR-based techniques, they have inherent disadvantages, such as high cost and requiring highly specialized equipment for the amplification and detection of the amplified products, and associated laboratory skills. These factors make PCR unsuitable in developing countries or resource-poor settings [12]. Thus, a rapid, simple cost-effective assay is needed to complement the current methods.

Recently, loop-mediated isothermal amplification (LAMP) has been used as a simple, highly specific method for amplifying DNA. The method is based on the autocycling strand-displacement DNA synthesis functions performed by the enzyme *Bst* DNA polymerase, where the target DNA is amplified greatly from a few copies of DNA in < 1 hour, requiring no special reagents under isothermal conditions [13, 14]. LAMP-positive products can also be confirmed by adding a fluorescent DNA intercalating dye or a metal indicator before the reaction, allowing observation with the naked eye; all steps, from amplification to detection, are conducted in one reaction tube. To date, LAMP has been successfully developed to detect *Entamoeba histolytica* [15] and *Acanthamoeba* [16] but not *N*. *fowleri*.

In the present study, we developed the first visual closed-tube LAMP specifically for the detection of *N*. *fowleri*, and successfully detected the organism in spiked water and clinical CSF samples. Our results indicate that this LAMP is sensitive, specific, and could be developed into an early diagnostic method for PAM, as well as an alternative tool in epidemiological surveys.

## Materials and Methods

### Ethics statement

The use of leftover CSF for making spiked samples was approved by the Ethics Committee of the Faculty of Tropical Medicine, Mahidol University (MUTM 2006–063) and the Ethics Review Committee for Research in Human Subjects, Ministry of Public Health, Thailand (Ref. No. 33/2550). A written informed consent form regarding the use of leftover CSF specimens for future research purposes was obtained from the patients. The CSF was completely anonymous and was not linked with the patients’ identification.

### Parasites and bacteria

*Naegleria fowleri*, *Naegleria gruberi*, *Acanthamoeba* spp., trophozoites, isolated from environmental samples from our previous study [17] and confirmed by morphology, culture, mouse pathogenicity and sequencing.

*Giardia duodenalis*—cysts, cultured and confirmed by PCR.

*Cryptosporidium parvum*—oocysts, cultured and confirmed by PCR.

*Entamoeba histolytica*—trophozoites, cultured and confirmed by PCR.

*Entamoeba coli*—cysts, detected by morphology.

*Toxoplasma gondii*—oocysts, kindly provided by Prof. Marie Laure Darde, University Hospital, Department of Parasitology, Biological Resource Centre for *Toxoplasma*, Limoges, France.

*Neospora caninum*—tachyzoites, cultured and confirmed by PCR.

*Blastocystis*—confirmed by sequencing.

*Enterocytozoon bieneusi*—confirmed by sequencing.

*Escherichia coli*—kindly provided by the Department of Microbiology and Immunology, Faculty of Tropical Medicine, Mahidol University.

*Cryptococcus neoformans*—kindly provided by Prof. Srisurang Tantimavanich, Department of Clinical Microbiology, Faculty of Medical Technology, Mahidol University.

*Mycobacterium tuberculosis*—strain H37Rv, kindly provided by the Central Chest Institute of Thailand.

### LAMP primer design

A set of five primers for LAMP were designed according to the published sequence of *N*. *fowleri* virulence-related protein (GenBank accession no. M88397) [18] using PrimerExplorer ver. 4 (http://primerexplorer.jp/elamp4.0.0/index.html). A forward inner primer (FIP), a backward inner primer (BIP), two outer primers (F3 and B3) and a loop primer (LB) were used for the LAMP. The primers were aligned and checked for specificity using NCBI GenBank and comparative genome Basic Local Alignment Search Tool (BLAST) analysis. The oligonucleotide primers were synthesized with High Affinity Purification by Bio Basic Inc. (Ontario, Canada). The sequences and lengths of the primers are shown in Table 1.

**Table 1:** Details of the primer set targeting *N*. *fowleri* virulence-related protein used for amplification in the LAMP assay.

Primers	No. of bases	Sequence (5’-3’)
F3	20	TGGATGGAGTAAGAGAGTTG
B3	25	TGAGTGTAGTTAATAATTCCTGTAC
FIP	46	GCAATGGATTGATTTGGAACGCAACAATGAAAGAAACTTTGCACCT
BIP	38	TTCCGTAGATTGGACGTCCATCCATCCATTTGGATCGG
LB	25	GCATTAGGAGTGAGAAGAAAGACTG

#### LAMP assay

The LAMP conditions were optimized with different parameters, including the concentrations of outer primers (0.1 to 0.4 μM), inner primers (1.2 to 2.0 μM), loop primer (0.2 to 1.0 μM), MgSO_4_ (5 to 10 mM), dNTPmix (1 to 2 mM), hydroxy naphthol blue (HNB) (80–160 μM), betaine (0 to 1 M), enzyme (2 to 10 U), assay temperature (60, 62, or 64°C) and incubation time (60, 90, or 120 min). Nine parameters, not including incubation time, were optimized one at a time and each optimization experiment was repeated three times. A non-template control (sterile water) as negative control and a positive control (DNA extracted from 100 *N*. *fowleri* trophozoites) were included for each LAMP run. The results were considered positive when the LAMP product showed a significant difference between positive amplification, i.e., when color changes from violet to sky blue; and negative amplification, which remains violet with the presence or absence of the ladder-like pattern bands after electrophoresis. On the basis of the above optimization steps, the final reaction mixture (25 μl) contained 0.2 M each of outer primer (F3, B3), 1.6 M each of inner primer (FIP, BIP), 0.8 M of loop primer (LB), 1X of supplied ThermoPol buffer, 8 mM MgSO_4_ (New England Biolabs, Ipswich, MA, USA), 1.4 mM dNTP mix (Thermo Scientific, Vilnius, Lithuania), 0.8 M betaine (Sigma-Aldrich, St. Louis, MO), 120 μM HNB (Sigma-Aldrich), 8 units of *Bst* DNA polymerase (New England Biolabs) with 2 μl total DNA as a template. Amplification was conducted at 64°C for 60 min and terminated at 80°C for 5 min.

#### Specificity of the LAMP primers

To investigate LAMP primer specificity, DNA templates isolated from organisms described earlier were subjected to LAMP. In addition, DNA extracted from CSF from non-PAM patients and healthy human blood were included in the test. The specificity test was repeated twice. Negative controls were included for all experiments.

### Determinations of lower limit of detection by LAMP and PCR in spiked CSF and water samples with pure cultures

The lower limit of detection of the LAMP assay was determined using known amounts of *N*. *fowleri* in pure cultures. The trophozoites were counted under a microscope with a hemocytometer and further serial 10-fold dilutions were prepared in phosphate buffered saline (PBS), ranging from 10^4^–1 cells. *N*. *fowleri* in each dilution was added into each 250 ml of water collected from a pond which was previously tested to be free of *N*. *fowleri* by microscopy and PCR, and 200 μl of pooled leftover CSF from non-PAM patients. CSF was obtained from HIV patients with neurological abnormalities. The water samples with cells were then centrifuged at 2,500 rpm for 10 min at 20°C as described by Puzon et al. [19]. Supernatant was discarded and samples resuspended in 1 ml of PBS, transferred to a 1.5 ml tube and reconcentrated by centrifugation at 10,000 rpm for 10 min at 20°C. Supernatant was then removed by pipette, before samples were resuspended in 200 μl PBS. Genomic DNA was extracted using a QIAamp DNA minikit (Qiagen, Hilden, Germany). LAMP sensitivity was compared with the conventional nested PCR method for *N*. *fowleri* [9]. Spiking experiments with different concentrations were repeated three times. In addition, another set of spiked water and CSF samples were subjected to DNA extraction using the heating method to compare the sensitivity obtained with DNA extraction by a commercial kit, to shorten the total completion time and to simplify the LAMP assay for the feasible diagnosis of field samples. The last dilution with all three samples testing positive was considered as the detection limit.

### DNA extraction and PCR assays

Genomic DNA was purified from the organisms listed above for specificity testing, the spiked dilutions for sensitivity test and the water samples for validating the LAMP, using a QIAamp DNA minikit (Qiagen, Germany) per manufacturer’s protocol and the DNA was eluted in 100 μl TE buffer. 2 μl of the DNA template was used in LAMP and PCR. The extracted DNA was stored at -80°C until use.

For DNA extraction by heating method, 200 μl of each spiked water dilution was heated directly at 95°C for 10 min without the centrifugation and resuspension steps. The samples were then centrifuged at 14,000 rpm for 10 min and 2 μl of the supernatant was used as the DNA template.

Classical nested PCRs from previously published primers targeting Mp2Cl5 sequence [9] were selected to compare and evaluate the sensitivity and efficiency of the LAMP. The forward primer, *Mp2Cl5*.for (5’-TCTAGAGATCCAACCAATGG-3’) and the reverse primer, *Mp2Cl5*.rev (5’-ATTCTATTCACTCCACAATCC-3’) were used in the first round of PCR amplification, which contained 1X *Taq* buffer, 2.5 mM MgCl_2_, 0.6 μM each of primer, 0.2 mM each of dNTP (Thermo Scientific, Vilnius, Lithuania), 2.5 units of *Taq* DNA polymerase (Thermo Scientific, Vilnius, Lithuania), and 2 μl of extracted DNA. Reactions were cycled 35 times with first denaturation at 95°C for 5 min, followed by denaturation at 95°C for 1 min, annealing at 65°C for 1 min and extension at 72°C for 2 min. The *Mp2Cl5*.for-in (5’-GTACATTGTTTTTATTAATTTCC-3’) and *Mp2Cl5*.rev-in (5’-GTCTTTGTGAAAACATCACC-3’) primers which amplified 110-bp fragment products were used for the nested PCR. The mixtures for the second PCR were the same as in the first round, except that the concentration of each primer was 0.5 μM. Two microliters of the PCR product from the first-round PCR were used in the second PCR. The LAMP and PCR products (7.5 μl) were analyzed by 2% agarose gel electrophoresis, stained with ethidium bromide, and observed under UV transillumination.

### Collection of environmental water samples for *N*. *fowleri* analysis

We conducted a survey for *N*. *fowleri* in Thailand to validate the applicability of the newly developed LAMP assay. Eighty water samples from different locations were collected from stagnant water around the houses and near the markets during the flooding crisis in Nakhon Pathom Province, Thailand, during November 2011. 30 water samples were collected from rivers, canals, rain (from a rainwater tank supplying a house), tap (from a public water distribution system), ground and underground water in Nan, Phayao, Kanchanaburi, Chantaburi, Trat, and Trang Provinces, Thailand, from January 2012 to December 2013 (S1 Fig). Samples were collected in sterile glass bottles and labeled with date, time, and place of collection. The water samples were processed in the manner described in US EPA Method 1623 with some modifications [20]. Briefly, 1,000 ml of water from each site were added to a sterile bottle and immediately sent to the laboratory. Each water sample was concentrated by multiple centrifugation steps (10 min at 3,500 rpm) and the pellet was resuspended in 1 ml of PBS, kept for DNA extraction using a QIAamp DNA minikit. Each sample was tested in triplicate by both LAMP and PCR assays.

All of the positive detection from environmental samples by LAMP were further tested for confirmation as *N*. *fowleri* by bidirectional sequencing of LAMP products [21]. To do this, 1 μl of LAMP product was used as template in a PCR reaction with primers that flank within the LAMP product of the *N*. *fowleri* virulence-related protein, F2 (5’- CAATGAAAGAAACTTTGCACCT-3’) and B2 (5’- TCCATCCATTTGGATCGG -3’) (0.3 μM each of primer), dNTPs (0.2 mM), *Taq* buffer (1X), MgCl_2_ (1.5 mM) and *Taq* DNA polymerase (1 unit) in final volume 25 μl. The reaction was run as follows: 2 minutes at 95°C, 35 cycles of 95°C 30 sec, 55°C for 30 sec, 72°C 30 sec, followed by 10 minutes extension at 72°C. The PCR product was run on an agarose gel, purified, and sequenced on an ABI3730xl DNA Analyzer (Applied Biosystems). The sequences were analyzed and identified using the MEGABLAST search program (http://www.ncbi.nlm.nih.gov) from the GenBank database.

### Statistical analysis

McNemar's test was used to test for differences between the LAMP and PCR detection rates. The test was 2-sided and exact probabilities were calculated. The agreement between LAMP and PCR was also calculated through the Kappa coefficient with 95% confidence level [22]. All analyses were conducted with SPSS for Windows 18.0 (SPSS Inc.).

## Results

### LAMP primer specificity

**Fig 1:**
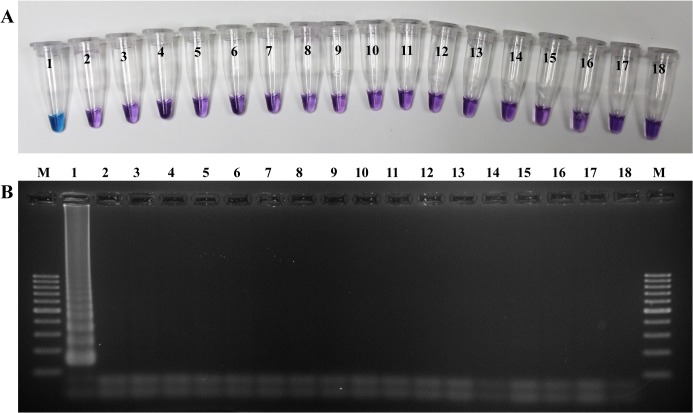
Specificities of the LAMP assay for the detection of *N*. *fowleri*. (A) Specificity of LAMP assay using HNB (note the sky-blue color for a positive sample). (B) Confirmation of results of the LAMP products using agarose gel (2%) electrophoresis. In panels A and B: M, 100 bp DNA Ladder (Thermo Scientific); 1, *N*. *fowleri*; 2, *N*. *gruberi*; 3, *Acanthamoeba* spp.; 4, *G*. *duodenalis*; 5, *C*. *parvum*; 6, *E*. *histolytica*; 7, *Entamoeba coli*; 8, *T*. *gondii*; 9, *N*. *caninum*; 10, *Blastocystis*; 11, *E*. *bieneusi*; 12, *Escherichia coli*; 13, *C*. *neoformans*; 14, *M*. *tuberculosis*; 15, CSF from non-PAM patients; 16, blood sample of healthy donor; 17, *N*. *fowleri*-free pond water; 18, no template control.

The specificity of the designed LAMP primers was examined by testing with genomic DNA of *N*. *fowleri*, *N*. *gruberi*, *Acanthamoeba* spp., *G*. *duodenalis*, *C*. *parvum*, *E*. *histolytica*, *T*. *gondii*, *N*. *caninum*, *Blastocystis*, *E*. *bieneusi*, *E*. *coli*, *C*. *neoformans*, and *M*. *tuberculosis*, as well as human genomic DNA and water collected from a pond which was previously tested to be free of *N*. *fowleri*. According to the results of the specificity test (Fig 1), only *N*. *fowleri* was detected as a positive color change (Fig 1A), which was confirmed by a ladder-like pattern on the agarose gel (Fig 1B), while DNA of other organisms and human DNA could not be amplified in the developed LAMP assay. These results demonstrated the specificity of the designed LAMP primer set for detecting the *N*. *fowleri* virulence-related protein gene.

### Comparison of LAMP and conventional PCR in spiked samples

**Fig 2:**
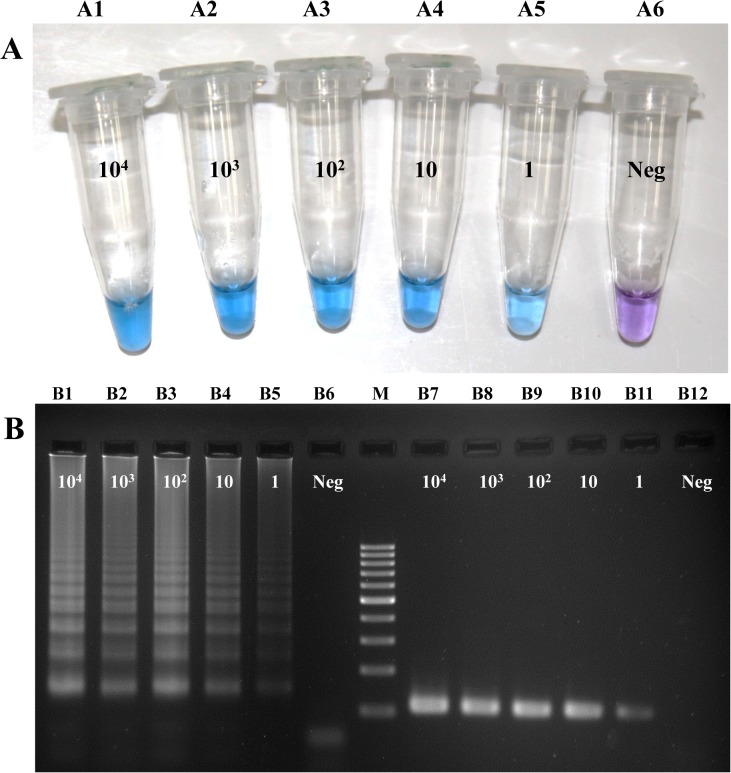
(A) The Lower limit detection of LAMP and PCR results in spiked water samples (Table 2A) was determined by making 10-fold dilutions from *N*. *fowleri* ranging from 10^4–^1 cells/250 ml of water, processed and extracted by QIAamp DNA minikit. Analytical sensitivity showed identical results in the LAMP (A1-A6) and PCR assays (B7-B12). (B) Electrophoresis results of the LAMP products from A1-A6 (B1-B6) and PCR products (110 bp) (B7-B12). M, 100 bp DNA Ladder; Neg, negative control.

To determine the analytical sensitivity of the LAMP assay, 10-fold dilutions of *N*. *fowleri* trophozoites from 1,000 to 1 cell(s)/250 ml of water and 200 μl of CSF, were used as templates for the LAMP and PCR experiments. The same dilutions were also used as templates for the PCR. In both techniques, the detection limit was 1 trophozoite in 250 ml of spiked water which is equivalent to 4 trophozoites in the field samples of 1,000 ml (Fig 2 and Table 2A) and 1 trophozoite in 200 μl of spiked CSF samples extracted by QIAamp DNA minikit. However, in the samples extracted using the heating method, the lower concentration of 1 trophozoite was detected by LAMP in two of three repeats in spiked water and all of three repeats in spiked CSF samples, compared with one of three repeats of 1 trophozoite in both spiked water and spiked CSF for PCR. This indicates that the LAMP assay has a higher sensitivity than PCR (Table 2B) under these conditions.

**Table 2:** Lower limits of detection of LAMP and PCR assays.

(A) Lower limit detection test of spiked samples with DNA extracted by QIAamp DNA minikit					
Sample type	Trophozoites/reaction				
	10^4^	10^3^	10^2^	10	1
Spiked water					
LAMP	+^*a*^	+	+	+	+
PCR	+	+	+	+	+
Spiked CSF					
LAMP	+	+	+	+	+
PCR	+	+	+	+	+
(B) Lower limit detection test of spiked samples DNA extracted by heating method					
Sample type	Trophozoites/reaction				
	10^4^	10^3^	10^2^	10	1
Spiked water					
LAMP	+	+	+	+	± (2/3)
PCR	+	+	+	+	± (1/3)
Spiked CSF					
LAMP	+	+	+	+	+
PCR	+	+	+	+	± (1/3)

^*a*^ +, triplicated assay showed all positive; ±, triplicated assay showed both positive and negative (positive number/test number);-, triplicated assay showed all negative.

### Performance of LAMP for *N*. *fowleri* detection in water samples

A total of 110 field water samples were collected from suburban and rural areas in central, northern, eastern, western, and southern Thailand, to validate the applicability of the LAMP for detection of *N*. *fowleri*, compared with PCR. The techniques reported similar results, where LAMP detected *N*. *fowleri* DNA in 5 of 80 samples (6.25%) and PCR detected 4 samples (5%) in flood water; 4 positive samples by PCR were also positive by LAMP (Table 3). In the other water types, 8 of 30 were found positive by LAMP (1 rain, 3 surface, 3 ground, and 1 tap water) and 6 samples were positive by PCR (3 surface, 2 ground, and 1 tap water). There were discrepancies for a total of 3 samples (1 flood in Nakhon Pathom Province, 1 rain and 1 ground in Nan Province). All LAMP positive detections were confirmed by sequencing. Proceeding bioinformatics analysis showed that the sequences of the LAMP products were perfectly (99–100%) matched with the virulence-related protein sequence (M88397) deposited in the GenBank database (data not shown). According to this data set, the overall detection rate between LAMP and PCR in water samples was not significantly different (2-sided McNemar's test, *P* = 0.25) (Table 3). Application of the Kappa coefficient test revealed a level of agreement = 0.855. A Kappa value > 0.6 is considered an index of substantial agreement, with a value > 0.8 indicating almost perfect agreement [23] (Table 4).

**Table 3:** Categories of water samples tested in this study with number *N*. *fowleri*-positive by LAMP and PCR.

Location and type of water (No. of sample)	Area characteristic	LAMP positive/examined	PCR positive/examined
Nakhon Pathom Province	Suburban		
- Flood (80)		5/80	4/80
Nan Province	Rural		
- Rain (6)		1/6	0/6
- Surface (4)		1/4	1/4
- Ground (1)		1/1	0/1
- Stream (1)		0/1	0/1
Phayao Province	Rural		
- Surface (3)		1/3	1/3
Kanchanaburi Province	Rural		
- Rain (2)		0/2	0/2
- Ground (2)		0/2	0/2
Chantaburi Province	Rural		
- Rain (2)		0/2	0/2
- Tap (2)		1/2	1/2
- Ground (1)		1/1	1/1
Trat Province	Rural		
- Tap (1)		0/1	0/1
- Ground (1)		1/1	1/1
Trang Province	Rural		
- Rain (1)		0/1	0/1
- Surface (2)		1/2	1/2
- Ground (1)		0/1	0/1
Total (110)*		13/110 (11.8%)	10/110 (9%)

*P value for McNemar's test = 0.25

**Table 4:** Agreement (Kappa) between the detection of *N*. *fowleri* by LAMP and PCR in water samples.

	No. of samples			
PCR result	LAMP positive	LAMP negative	Total	Kappa value*
Positive	10	0	10	0.855
Negative	3	97	100	
Total	13	97	110	

*P <0.001

## Discussion

LAMP is a highly specific, sensitive, rapid, and reproducible gene amplification assay. The main advantages of LAMP over other techniques are that it is easy to perform, can produce a lot of specific amplification products at a constant temperature without the need for expensive equipment, and the amplification reaction result can be determined visually by the naked eye [14]. Such a test could provide a useful diagnostic tool in a clinical laboratory or a field study, particularly in resource-poor countries. LAMP has been widely used for the detection of protozoan infections [15, 16, 24], but not for *N*. *fowleri*. To the best of our knowledge, this is the first report developing LAMP as a convenient tool for *N*. *fowleri* detection.

Possibly because PAM is a rare disease, only a few of the potential DNA targets of the parasite have been explored. Until now, the most popular DNA target allowing for the discrimination of *Naegleria* spp. in environmental samples was the complete ribosomal internal transcribed spacer region (ITS) [4]. Another commonly used target is the 18S rRNA that was applied to the detection of *Naegleria* spp. in formalin-fixed paraffin embedded brain tissue using real-time PCR [11] and in CSF using multiplex real-time PCR [10]. However, for the specific detection of *N*. *fowleri*, PCR targeting a cloned fragment of *N*. *fowleri* (MCM strain) [25], Mp2Cl5 [9] and virulence-related genes [26] have been described. Successful amplification of the LAMP relies on the specificity of the designed primers. With respect to the recently described genes, previous efforts to design and develop LAMP based on the Mp2Cl5 met with limited success, because of unsuccessful LAMP primer designs by Primer Explorer ver. 4 software. Although the program could generate primer sets for the Mp2Cl5 gene, these primers exhibited low sensitivity or unspecific amplification results. This is probably due to the shorter length sequences of the genes (650 bp in Mp2Cl5 vs. 1577 bp in virulence-related gene), which make it difficult to get good primers for use in LAMP [27]. In contrast, we successfully amplified the virulence-related gene [18] by LAMP with primers designed by the software. This gene is a robust diagnostic target because it is specific for *N*. *fowleri*, which is important for diagnostic identification; if primers are well-designed, it will allow for the highly specific identification of *N*. *fowleri* from other *Naegleria* spp. (*N*. *lovaniensis*, *N*. *australiensis*, *N*. *gruberi*, *N*. *andersoni*, and *N*. *jadini*) and other protozoa, as described previously [26].

The specificity of the LAMP primers was tested by screening different DNAs derived not only from *N*. *gruberi*, but also from different parasites which could be found in water samples (including *Acanthamoeba*, *Giardia*, *Cryptosporidium*, *Escherichia*, *Entamoeba*, etc.) and other pathogens that cause CNS diseases and symptoms similar to those observed in PAM (*Toxoplasma*, *Cryptococcus*, *Mycobacterium*) (see Fig 1). Although the LAMP was not tested with closely related animal model pathogens and thermophilic species of *Naegleria*, *N*. *australiensis* and *N*. *italica*, and the commonly found thermophilic species, *N*. *lovaniensis*, it has extremely high specificity, as the reaction only occurs when the six distinct regions within the DNA target are recognized by the four primers [28]. Moreover, sequencing results of all positive LAMP results from field water samples (Table 4) confirmed the ability of primers to specifically detect *N*. *fowleri* alone. Based on the data described above, our newly developed LAMP method was highly specific for *N*. *fowleri*. However, it has been reported that LAMP can result in non-template amplification due to several factors, including the nature of LAMP primers and the reaction conditions, which include high concentrations of primer and magnesium [29]. Therefore, it may be necessary to optimize the system to ensure that the LAMP meet the requirements of specificity and sensitivity.

This study also demonstrated that the LAMP method described here is highly sensitive in spiked water and CSF samples. The detection limit of the PCR was 5 pg of *N*. *fowleri* DNA or 5 intact *N*. *fowleri* amoebae in spiked tap water and water collected in rivers and lakes [9]. In real-time PCRs, the detection limits were 1 copy of the Mp2Cl5 DNA sequence [6] and approximately 3 cell equivalents in water samples [30]. Therefore, the detection of target DNA by LAMP compared with detection by previous molecular methods was at least equivalent (Fig 2) or more sensitive, which was confirmed by the results in Table 2B showing that the detection limit of LAMP was as sensitive as the currently used assays for the detection of *N*. *fowleri*.

Many rapid detection assays have recently been developed and optimized to overcome conventional culture and microscopy techniques to detect *N*. *fowleri* in clinical and environmental samples. Many other studies also reported the superior sensitivity of real-time PCR over PCR, immunohistochemistry, or culture [5, 8, 11, 30]. The total reaction time (60 min) used for LAMP assay reported here was similar to real-time PCR assays developed by others [6, 8, 10, 11], but markedly faster than conventional PCRs [9, 25, 26] by at least 2–3 hours, therefore significantly reducing assay time. Although real-time PCR assays are powerful, sensitive, and efficient tools for detecting *N*. *fowleri*, the requirement of an expensive thermal cycler has limited their application for field diagnostic tests. In contrast, the LAMP test requires only a dry block or water bath for the amplification, which is the main benefit over real-time PCR. Moreover, the possibility of using heat-processed samples without compromising sensitivity eliminates the need for a conventional DNA extraction step, requires less equipment and reduces the likelihood of sample contamination, shortening the time taken compared with PCR-based techniques (10 min by heating method vs. 20 min by DNA extraction kit or at least 2 h by standard Phenol/Chloroform DNA extraction) [31]. However, a disadvantage of the LAMP with heating method is that the DNA cannot be stored reliably long-term. [32]. Another advantage of the LAMP method is the use of HNB, which aids in monitoring the reaction. It can be added to the reaction mixture before incubation so that amplification is completed in a closed-tube system and reduces the risk of carry-over contamination in the post-PCR process [33]. When compared with other visible endpoint detection methods, such as visualization of turbidity, the judgment of positive and negative results using HNB as a colorimetric endpoint indicator can be easily distinguished by color changes (Fig 1) and this intercalating dye is also stable in solution for months [34].

In this study, 13 of the environmental samples were positive by LAMP and 10 by PCR. The detection rates of the two assays did not differ (*P* >0.05) and there was excellent agreement between assays (Kappa value >0.81) [23], indicating that LAMP and PCR are similarly capable of detecting *N*. *fowleri* in water. Of 80 floodwater samples tested, 5 and 4 were positive by LAMP and PCR, respectively. *Naegleria* spp. and *Acanthamoeba* spp. were identified by culture during the flood disaster in Chiang Mai, Thailand [35]. To date, this is the first report of the direct identification of *N*. *fowleri* in flood water by molecular methods. In many areas of Thailand, the flood rose so rapidly that countless people could not evacuate in time and had no way to escape. Some made it to rooftops, others tried to find their way to a dry place by swimming through the floodwaters [36]. In addition, many children took the opportunity to play and engage in water-related activities during this time. Although there is no mention of PAM as a result of flooding, in a situation like this, identifying *N*. *fowleri* quickly would help to indicate that clinicians should be aware of PAM and include it in the differential diagnosis of meningoencephalitis [37].

When the non-flood water was examined, again, higher numbers of *N*. *fowleri* were identified compared with PCR, with 1 ground and 1 rain positive samples for LAMP, which were negative for PCR. The inconsistent results in this study are likely due to the lower detection limit of the LAMP as shown in the tested spiked water samples. Another contributing factor could be PCR inhibitors from the natural characteristics of environmental samples, which could affect the *Taq* DNA polymerase used in conventional PCR but may not affect the *Bst* polymerase used in LAMP [15, 38].

Unfortunately, data from previous studies conducted in Thailand only showed the presence of *Naegleria* spp. in environmental water from canals (1.4%) [39], natural or man-made lakes (28.6%) [40], hot springs (35.3%) [41], and thermal water ponds (19%) [42]. Only 1 study revealed the presence of *N*. *fowleri* in 10% of water samples (warm and fresh ponds in industrial areas) as characterized by morphology, pathogenicity and pathology in rats [43]. The higher prevalence of *N*. *fowleri* found in non-flood water samples in this study (26.6%) may be due to the larger volume of collected water (1,000 ml vs. 300 ml in the previous study [43] or the higher sensitivity of the LAMP technique (Table 2B).

*N*. *fowleri* has been isolated from soil, domestic water supplies, well water, artificially heated industrial water sources, chlorinated swimming pools, natural hot springs and hot tubs [7, 44–46]. The primary sources of water for many Thai citizens in rural areas are surface and ground-water. Untreated domestic sewage and industrial waste-water have increased in the surface water bodies [43]. It is unclear how *N*. *fowleri* were found in the tab and rain water in this study. However, once introduced, they were able to colonize even though there is a small risk associated with these kinds of water. Infection with *N*. *fowleri* was thought to be acquired through modes other than conventional swimming or diving in ponds and lakes. Immersion of the head in a trough of water in a school playground, total immersion in bathwater and playing in a warm muddy puddle after rain have been described as sources of infection with the amoeba [7]. *N*. *fowleri* have been detected in studies of household plumbing and related surfaces and free-living amoeba are detected routinely in household plumbing and appurtenances. The Louisiana homes neti pot deaths and the play slide death tested positive for *N*. *fowleri* at various points in the premise plumbing [47–48].

One limitation to this study was the lack of real PAM specimens since cases are very rare. Although the LAMP here demonstrated a good lower detection limit in spiked CSF, the practicality of the technique is unclear without testing using clinical specimens [12]. Therefore, more studies are needed to demonstrate the feasibility of the application of the highly sensitive and specific LAMP assay to clinical specimens such as CSF, tissues or nasal discharges as well as to other types of environmental samples e.g. soil, dust etc. Moreover, the LAMP assay could be further developed into a real-time quantitative format [38], to assess the risk of PAM in association with the number of parasites.

In conclusion, for the first time we successfully developed a LAMP assay with significant potential for development into a field-based diagnostic system. It could be used away from the laboratory and in environments where access to expensive equipment is not possible, since it requires minimal equipment for both DNA extraction and subsequent LAMP analysis. However, further study of the LAMP method using real clinical specimens and a larger number of environmental samples will be needed to validate the assay. Such a test might help to apply the appropriate treatment in clinical practice, or for use in large-scale epidemiological studies in the future.

## Supporting Information

S1 Fig Map showing the sampling areas from different water sources in this study. (TIF)
