# Supplementary figures and images for: Development of a Rapid, Simple Method for Detecting Naegleria fowleri Visually in Water Samples by Loop-Mediated Isothermal Amplification (LAMP)
Source: PLoS One. 2015 Mar 30;10(3):e0120997. doi: 10.1371/journal.pone.0120997 (PMC4379150; doi:10.1371/journal.pone.0120997)

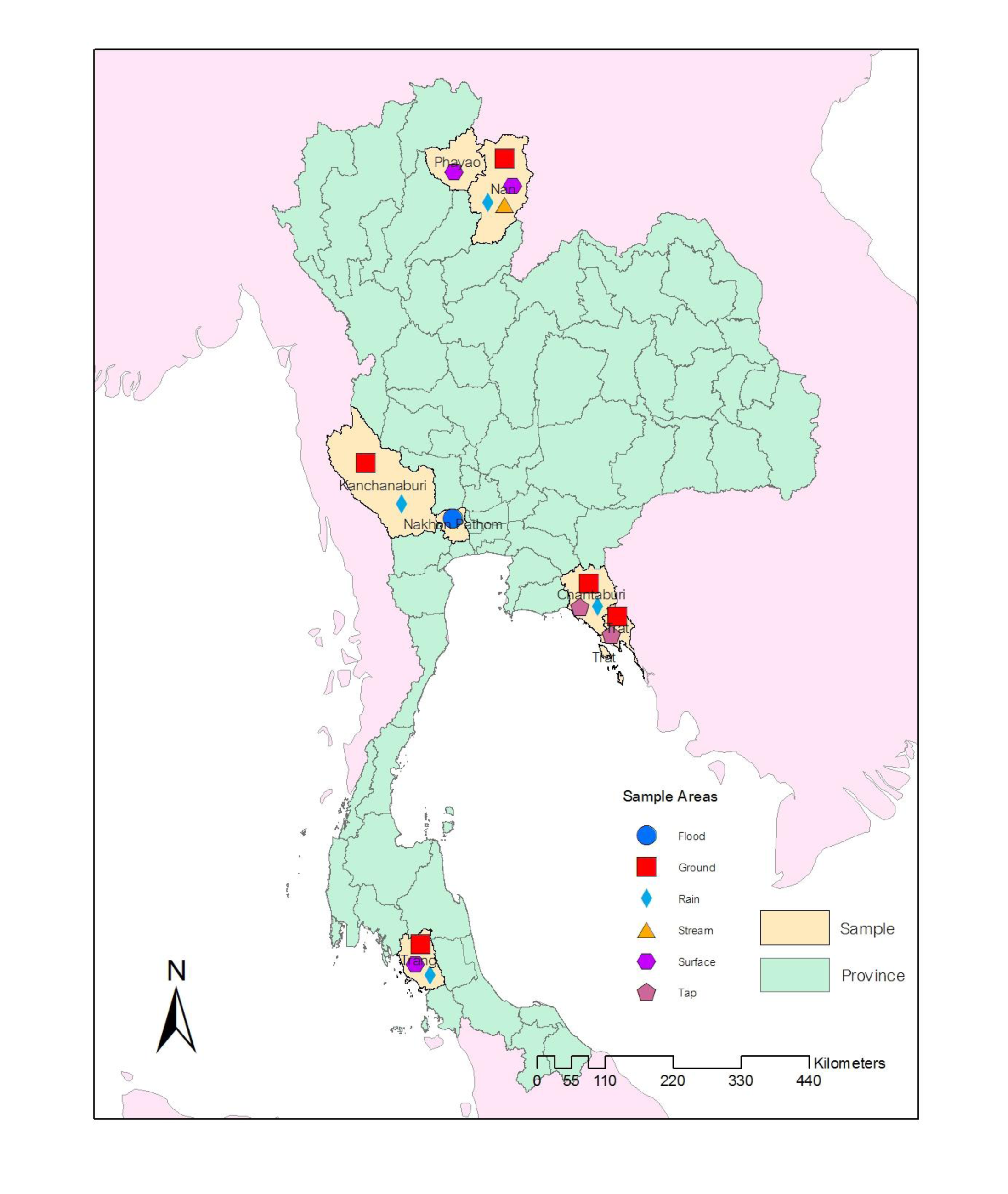

Supplement: S1 Fig — (TIF) [file pone.0120997.s001.tif]
